# Supplementary material for: Learning curve and surgical outcome of robotic assisted colorectal surgery with ERAS program
Source: Sci Rep. 2022 Nov 29;12:20566. doi: 10.1038/s41598-022-24665-w (PMC9709162; doi:10.1038/s41598-022-24665-w)
Supplement: Supplementary file 1 — Supplementary Information 1. [file 41598_2022_24665_MOESM1_ESM.zip › LSC Raw data-Ñ[▒K/133/H_ñw╝╨░O▒Kñσ.pdf]

正式摘要(電子簽章：000000223819928)

W83 / W83 ☐ 英文標題(注意:無英文姓名-請補病人基本檔!) ☐【讀取】憑證

臺中榮民總醫院

出院病歷摘要

轉入號： 轉出號：

[redacted]

(8)流水編號	
(9)入院日期	110/08/18 (2021) 直腸外科 W83 - 065
(10)轉科日期	
(11)出院日期	110/08/24 (2021) 住院天數計 6 日

診斷

- 入院：1. Adenocarcinoma of A-colon, cT3N2M0
- 出院：1. Adenocarcinoma of A-colon, s/p laparoscopic right hemicolectomy on 2021/08/19, pT2N0, stage I

主訴

- Intermittent diffused abdominal pain for a month

病史

- This is a 38 year-old man, denied any systemic disease before.
  This time, he had intermittent diffused abdominal pain for about a month. Accompany with bowel habit change (diarrhea, constipation). CEA: 2.04 CA-199: 20.4 No hepatitis B or hepatitis C. Denied any body weight loss recently. Due to those discomfort, he went to OPD for help. Colonscope with biopsy was done, and pathological report showed A-colon adenocarcinoma with moderately differentiated. Under impression of A-colon adenocarcinoma, he was admitted for right colectomy.

體檢發現

-
  - General appearance :
    - easy looking , conscious clear , without cardiopulmonary distress
    - Vital signs自動帶入date 體溫(BT) 脈搏(P) 呼吸(R) 血壓(BP) 身高(BH) 體重(BW) BMI
      20210818 36.1℃ 85次/分 18次/分 134/85mmHg
      20210819 98.3Kg
    - Vital signs: B.P. 134/85 mmHg , P.R. 85 /min , R.R. 18 /min , B.T. 36.1 ℃
  - HEENT :
    - Eyes: no anemic conjunctiva, no icteric sclera, normal EOM
    - ENT: no otorrhea, no rhinorrhea, no erythematous mucosa, no injected tonsil, no deformity
  - Neck: supple, freely movable, trachea midline,no deformity, no engorged jugular vein , no palpable thyroid or lymph node enlargement
  - Chest:
    - Inspection: Grossly normal, symmetric and fully expansion, no accessory muscle used, no operation scar, no spider angiomata, no gynecomastia
    - Palpation: no palpable mass or nodule, no subcutaneous emphysema, no tenderness
    - Ausculation: clear breathing sound
  - Heart:
    - Palpation: no thrill, no heave
    - Percussion: normal heart size
    - Ausculation: regular heart beat, normal S1, S2, no gallop, no murmur
  - Extremities: No deformity, full ranged of motion without limitation, no clubbing finger or nail cyanosis, no pitting edema, no ulcer or pigmentation, no obvious skin lesion, no varicose vein, no bone, joint or muscle tenderness

  CRS CONDITION
  - Karnofsky performance status: (100) Normal, no complaints, no evidence of disease

-
  - PE of Abdomen:
  - CEA: 2 ng/ml
  - Imaging Studies:
  - Colonoscope report: A-colon adenocarcinoma
  - Figures:
    1. colon & rectum

手術日期及方法

- 手術日期及方法20210819 Laparoscopic right colectomy and anastomosis

住院治療經過

- After admission, routine preoperative prepare was done, he underwent laparoscopic right hemicolectomy with anastomosis on 2021/08/19. After surgery, he had regained conscious and tolerated well with oral intake. Under his relatve stable condition, he was discharged on 08/26. .

合併症

- Nil

一般檢查

- 累積報告 :

DATE	TIME	ALB	TP	BIL.T	BIL.D	ALKP	AST	ALT	LDH	AMY	LIPASE	NH3	Ferritin	UA
1100818	1028	4.4		0.4	0.1	106	13	9	120					
1100819	2017	3.7		0.4	0.1		11	9						
1100823	0906							5						

DATE	TIME	WBC	RBC	HGB	HCT	MCV	PLT	BLST	NEUT	BAND	SEG	LYM	MON	EOS	BAS
1100818	1028	8100	4.82	12.0	37.2	77.2	426		67.6			23.8	6.2	1.9	0.5
1100819	2017	18530	4.37	11.0	35.2	80.5	359		95.7			2.8	1.2	0.1	0.2
1100823	0913	7460		9.8	31.7		328		63.2			25.5	7.5	3.4	0.4

DATE	TIME	NA	K	CL	BUN	CREAT	eGFR	CA	Mg	P	CRP	ESR	PCT	D-Dimer	LACTATE
1100818	1028	140	3.9		17	1.25		8.7							
1100819	2017	139	4.0		10	1.12	77.99	8.0							
1100819	2018														12.1
1100823	0906	141	3.7		6	1.05	84.02	8.0							

DATE	TIME	GLUCOSE	HbA1C	CHOL	TG	HDL	LDL	KETONE	CK	CKMB	TRO-T	PT-P	APTT-P	NT-pro BNP
1100818	1028	103										9.8	27.8	

特殊檢查

病理報告

- 活體組織切片病理報告　手術日期：**110/08/19**　病理號：**S1025777**

  診斷醫師:王任卿 報告日期:110/08/26
  Pathologic diagnosis:
  1. Colon, terminal ileum, and appendix, lapa right hemicolectomy ---
  a. Adenocarcinoma, moderately differentiated, of ascending colon.
  Tumor invaded to muscularis propria. The appendix, ileocecal valve,
  bilateral cut ends and mesocolic lymph nodes (0/41) are free of
  tumor. b. Reactive papillary mesothelial hyperplasia over mesocolon.
  2. Lymph node, 213, dissection --- Unremarkable (0/12).
  Ancillary study for diagnosis:
  1. IHC stains for calretinin (+), BAP-1 (preserved) and MTAP
  (preserved) done on section E.
  2. IHC stains for MSH2 (preserved), PMS2 (preserved) and MSH6 (loss)
  done on section G.
  Prognostic and predictive factor:
  1. Tumor size: 5.6x3 cm.
  2. Depth of invasion: Tumor invades to muscularis propria.
  3. Histologic grade: G2: Moderately differentiated.
  4. Angiolymphatic invasion: Present.
  5. Perineural invasion: Not identified.
  6. Tumor deposits (discontinuous extramural extension): Not
  identified.
  7. Macroscopic tumor perforation: Not identified.
  8. Surgical Margins: a) Proximal Margin: Uninvolved by invasive

- carcinoma, distance of tumor from margin: 13.5 cm. b) Distal Margin: Uninvolved by invasive carcinoma, distance of tumor from margin: 6 cm. c) Radial or Mesenteric Margin: Uninvolved by invasive carcinoma, distance of tumor from margin: 2 cm.
  9. Lymph node status: Uninvolved (0/53).
  10. Extranodal involvement: Not identified.
  11. Tumor regression grading S/P CCRT: No known presurgical therapy.
  12. Type of polyp in which invasive carcinoma arose: Not identified.
  13. Additional pathologic findings: Not identified.
  14. Pathological TNM Stage: pT2N0(According to the eighth edition, American Joint Committee on Cancer Staging Guidelines for Tumors).
  15. TNM descriptors: Not applicable.
  Gross description:
  The specimen consists of 1) a segment of colon, 8.6 cm in length, and 4.5 cm in proximal circumference, 3 cm in distal cut end circumference, and a segment of terminal ileum, 12 cm in length, and 3 cm in cut end circumference. An unremarkable appendix, 5x0.6 cm, is noted. A gray tan firm annular elevated and centrally ulcerative tumor, 5.6x3 cm, is noted, 6 cm to the nearest cut end. On cross section, the tumor invaded to the muscle layer with 20 mm to the radial margin grossly. The appendix, ileocecal valve, and both cut ends are free of tumor. 2) "LN213", 2 pieces, up to 4.7x2x0.7 cm.
  Representative part for section: A) proximal cut end B) distal cut end C) ileocecal valve D) appendix E-G) tumor H) colon J) specimen 2 K-P) lymph nodes.
  Note:
  1. The interpretation of MMR panel and recommended studies are as below: (1) No loss of nuclear expression of MMR proteins: low probability of microsatellite instability-high (MSI-H) . (2) Loss of nuclear expression of MLH1 and PMS2: testing for methylation of the MLH1 promoter and/or mutation of BRAF is indicated (the presence of a BRAF V600E mutation and/or MLH1 methylation suggests that the tumor is sporadic and germline evaluation is probably not indicated; absence of both MLH1 methylation and of BRAF V600E mutation suggests the possibility of Lynch syndrome, and sequencing and/or large deletion/duplication testing of germline MLH1 may be indicated). (3) Loss of nuclear expression of MSH2 and MSH6: high probability of Lynch syndrome (sequencing and/or large deletion/duplication testing of germline MSH2 may be indicated, and, if negative, sequencing and/or large deletion/duplication testing of germline MSH6 may be indicated). (4) Loss of nuclear expression of MSH6 only: high probability of Lynch syndrome (sequencing and/or large deletion/duplication testing of germline MSH6 may be indicated). (5) Loss of nuclear expression of PMS2 only: high probability of Lynch syndrome (sequencing and/or large deletion/duplication testing of germline PMS2 may be indicated). (6) Please note: There are exceptions to the above IHC interpretations. These results should not be considered in isolation, and clinical correlation with genetic counseling is recommended to assess the need for germline testing.
  2. This case has been peer reviewed by two doctors.
  #T-59420_2 #M-81403_2 2 1105 00000F
  #T-C4560_2 #M-09183_2 0 1105 000000
  #T-59200_2 #M-09183_2 0 1105 000000

其它

出院時情況

- 3.改門診治療

出院指示

藥名	劑量	劑量單位	用法	頻次	數量	數量單位	現況	天數	單位
(口)Takepron OD tab 30mg	1	TAB	PO	QDAC	7	TAB	TH	7	天
Econ tab 40mg	1	TAB	PO	QID	28	TAB	TH	7	天
Mag. Oxide tab	2	TAB	PO	TID	42	TAB	TH	7	天
Ultracet tab	1	TAB	PO	Q6HPRN	28	TAB	TH	7	天

出院計劃

- 門診日期
    - date on 2021/08/31
- 衛教 :一、飲食攝取：1.少量多餐。2.禁食刺激性食物，如太辣、太腥，大量飲酒及抽太多煙、濃茶和咖啡。3.多攝取高蛋白食物，以加速傷口修復。如：新鮮瘦肉、魚、蛋等。4.並減少吃產氣食物可避免脹氣。如：牛奶、豆類、地瓜、蛋、洋蔥。二、術後活動：1.三個月內不可從事下列活動：騎馬、騎腳踏車、劇烈的跳舞、久坐及開車，以免骨盆充血。2.手術後６？８週內避免提超過5KG之重物，若提物品可使用腹托或束腹帶以免震動所引起之疼痛。3.依個人的體力採漸進增加活動量，可先採輕工作，如無不適，體力許可再增加活動量。三、傷口護理：1.保持傷口清潔、乾燥，若有不慎弄濕傷口，可用清潔的毛巾或紗布拭乾。2.每日觀察傷口，是否有紅、腫、熱、痛，或不正常的分泌物。四、居家服藥：1.帶回的藥物，注意藥袋上的服用劑量、時間及注意事項。2.服用軟便劑若解便正常或有腹瀉現象時應停藥或減量。 五、出院後，若有下列情況需立即返院檢查：1.高燒不退。2.腹部疼痛厲害或嚴重腹脹嘔吐。3.傷口流血不止，或是有不正常之分泌物4.有其它問題，請與大腸直腸外科相關人員聯絡 TEL: 04-23592525 轉5161-3 或 6051, 6052
- OPD f/u

主治醫師 2062F 蔣鋒帆 / 住院醫師 2336L 洪浚嚴

正式摘要(電子簽章：000000223819928)

W83 / W83

doctorRank:B
